# Proinflammatory Cytokines Stimulate Mitochondrial Superoxide Flashes in Articular Chondrocytes *In Vitro* and *In Situ*

**DOI:** 10.1371/journal.pone.0066444

**Published:** 2013-06-19

**Authors:** Yongxing Cao, Xin Zhang, Wei Shang, Jiejia Xu, Xianhua Wang, Xiaoqing Hu, Yingfang Ao, Heping Cheng

**Affiliations:** 1 Institute of Sports Medicine, Peking University Third Hospital, Beijing, China; 2 State Key Laboratory of Biomembrane and Membrane Biotechnology, Institute of Molecular Medicine, Peking University, Beijing, China; Ohio State University, United States of America

## Abstract

**Objective:**

Mitochondria play important roles in many types of cells. However, little is known about mitochondrial function in chondrocytes. This study was undertaken to explore possible role of mitochondrial oxidative stress in inflammatory response in articular chondrocytes.

**Methods:**

Chondrocytes and cartilage explants were isolated from wild type or transgenic mice expressing the mitochondrial superoxide biosensor - circularly permuted yellow fluorescent protein (cpYFP). Cultured chondrocytes or cartilage explants were incubated in media containing interleukin-1β (10 ng/ml) or tumor necrosis factor-α (10 ng/ml) to stimulate an inflammatory response. Mitochondrial imaging was carried out by confocal and two-photon microscopy. Mitochondrial oxidative status was evaluated by “superoxide flash” activity recorded with time lapse scanning.

**Results:**

Cultured chondrocytes contain abundant mitochondria that show active motility and dynamic morphological changes. In intact cartilage, mitochondrial abundance as well as chondrocyte density declines with distance from the surface. Importantly, sudden, bursting superoxide-producing events or “superoxide flashes” occur at single-mitochondrion level, accompanied by transient mitochondrial swelling and membrane depolarization. The superoxide flash incidence in quiescent chondrocytes was ∼4.5 and ∼0.5 events/1000 µm^2^*100 s *in vitro* and *in situ*, respectively. Interleukin-1β or tumor necrosis factor-α stimulated mitochondrial superoxide flash activity by 2-fold *in vitro* and 5-fold *in situ,* without altering individual flash properties except for reduction in spatial size due to mitochondrial fragmentation.

**Conclusions:**

The superoxide flash response to proinflammatory cytokine stimulation *in vitro* and *in situ* suggests that chondrocyte mitochondria are a significant source of cellular oxidants and are an important previously under-appreciated mediator in inflammatory cartilage diseases.

## Introduction

Articular cartilage is an avascular connective tissue that contains only one specialized cell type, the chondrocyte, which is anchored in the dense extracellular matrix and is supplied with oxygen and nutrients by diffusion from surrounding tissues, principally, from the synovial fluid [1]. The entire chondrocyte metabolism operates under a low oxygen tension environment with the majority of the cell’s energy requirements being fulfilled by glycolysis that occurs in the cytoplasm [2]. Mitochondrial oxidative phosphorylation produces only a fraction of the total cellular ATP [3]. It has been reported that chondrocytes normally do not contain abundant mitochondria [4], and mitochondria mediated pathogenesis has not been sufficiently studied in models of cartilage diseases.

It has been reported that oxygen tension is 3.3–9.6 kPa in synovial fluid [5] and decreases with increasing distance from the cartilage surface to 1–1.25 kPa [6], [7]. A modeling study showed that oxygen tension in normal cartilage is not likely to fall to 1 kPa except under abnormal conditions [8]. In comparison, the median oxygen tension in systemic arterioles is ∼7 kPa and falls to ∼3–4 kPa in precapillary arterioles and capillaries [9]. Thus, chondrocytes, especially those in the superficial zone, are subjected to similar oxygen tension as that for the cells in blood-supplied tissues. Although chondrocyte energy metabolism is dominantly glycolytic rather than through oxidative phosphorylation, it has recently been shown that mitochondrial respiration supplies the oxidants critically needed for setting the redox balance for normal glycolytic ATP synthesis [10]. Studies in cartilage pathological animal models indicate that early cartilage degeneration begins in the superficial layer extending to the middle and deep zones with increasing denaturation [11]. Therefore, mitochondria, the major cellular consumer of oxygen, are very likely to play important physiological roles in articular chondrocytes, and more importantly, mitochondrial dysfunction has been reported to be involved in the process of cartilage degeneration [12]–[14].

In addition to ATP production, mitochondria have a plethora of physiological and pathological functions such as maintaining redox balance and committing apoptosis in different cell types and cellular contexts [15]. The mitochondrial respiratory chain is a major source of reactive oxygen species (ROS) [12], [16]. Increasing evidence suggests that ROS are not only deleterious agents involved in cartilage degradation but that they act as indispensable factors in intracellular signaling mechanisms [17]. Interestingly, we know little about mitochondrial ROS production in chondrocytes. Two previous studies have suggested that mitochondrial dysfunction induces an inflammatory response in cultured chondrocytes [13] and amplifies the effect of proinflammatory cytokine-induced inflammation through ROS production and NF-κB activation [14], but these results were based on experiments in cultured chondrocytes that did not examine the influence of proinflammatory cytokines on mitochondria. The aim of the present study was to visualize mitochondrial distribution and dynamics in living chondrocytes *in vitro* and *in situ* and explore chondrocyte mitochondrial ROS production under physiological and pathological conditions.

## Materials and Methods

### Ethics Statement

This study was carried out in strict accordance with the recommendations in the Guide for the Care and Use of Laboratory Animals of the National Institutes of Health. All the protocols were approved by the Institutional Animal Care and Use Committee (IACUC) of Peking University (permit number, IMM-ChengHP-14), which is accredited by AAALAC International.

### Generation of Pan-tissue Mitochondrially Targeted Circularly Permuted Yellow Fluorescent Protein Transgenic Mice

Recently we reported a serendipitous finding that a circularly permuted yellow fluorescent protein (cpYFP) is a novel genetically encoded indicator highly selective for superoxide anions, with sensitivity and kinetic properties suitable for real-time tracking of superoxide production [18]. To measure mitochondrial superoxide signals *in vivo*, we generated pan-tissue mitochondrially targeted cp-YFP (mt-cpYFP) transgenic mice in a C57BL/6 background. Full-length mt-cpYFP DNA was cloned into the XhoI site of a pUC-CAGGS vector downstream of a chicken β-actin promoter. The construct was then linearized with Spe1 and BamH1 in sequence, and was purified and microinjected into fertilized eggs from CBA/B6 mice. Compared with the first generation transgenic mice described previously [19], these new transgenic mice displayed more stable and uniform expression of the fluorescent indicator among different cells and tissues. The transgenic mice were kept on a 12-h light cycle; food and water were provided *ad libitum*.

### Isolation and Culture of Mouse Chondrocytes

Articular cartilage was obtained from the hip and knee joints of 4-week-old wild type (WT) C57BL/6 mice or mt-cpYFP transgenic mice killed by cervical dislocation. Cartilage slices were chopped and incubated for 4 h at 37°C in Dulbecco’s modified Eagle’s medium (DMEM, Life Technologies) containing 2 mg/ml type II collagenase (Sigma-Aldrich). The digested tissue was filtered with a nylon filter and the resulting cell suspension was centrifuged at 200×g for 5 min. The chondrocytes were then suspended in complete medium and cultured in a 5% CO_2_ incubator at 37°C. Subcultures were performed with a 0.25% trypsin-EDTA solution, and first passage chondrocytes were seeded into 35 mm dishes (Corning) for confocal imaging.

### Confocal Imaging of Cultured Chondrocytes

The chondrocytes were washed with Tyrode’s solution containing 137 mM NaCl, 20 mM HEPES, 10 mM D-glucose, 5.4 mM KCl, 1.2 mM MgCl_2_, 1.2 mM NaH_2_PO_4_, and 1.8 mM CaCl_2_ at pH 7.4 (adjusted with NaOH) and were imaged by either an inverted (Zeiss LSM 710) or an upright confocal microscope (Zeiss LSM 700). Real-time images were acquired using a 40×, 1.3 NA oil-immersion objective in the inverted configuration or a 40×, 1.0 NA water-immersion objective in the upright configuration at a sampling rate of 1.0 frame/s. Dual excitation imaging of mt-cpYFP was achieved by alternating excitation at 405 and 488 nm and collecting emission at >505 nm. Tri-wavelength excitation imaging of mt-cpYFP and tetramethylrhodamine methyl ester (TMRM, Molecular Probes) was achieved by tandem excitation at 405, 488, and 543 nm, and the emission was collected at 515–550, 515–550 and >560 nm, respectively.

### Confocal and Two-photon Excitation Imaging of Chondrocytes *in situ*

WT or mt-cpYFP transgenic mice were killed by cervical dislocation and the lower limbs were disconnected from the trunk at the hip joint. The skin and soft tissues were removed to isolate the femur. After gluing the body of the femur to the bottom of a 35 mm plastic culture dish with Vaseline, the dish was filled with Tyrode’s solution. A Zeiss LSM 710 NLO confocal microscope was used to detect TMRM staining to determine mitochondrial distribution in intact cartilage, with a 40×, 1.1 NA water-immersion objective, and excitation wavelength of 850 nm, and emission collection from 575 to 620 nm. A Zeiss LSM 700/710 confocal microscope was used to detect chondrocyte superoxide flashes in the superficial layer *in situ*.

### Image Processing and Superoxide Flash Analysis

Digital time-series images and Z-stack images were analyzed using custom-developed programs written in Interactive Data Language (IDL, Research Systems). Motion artifacts were largely corrected by image processing such that the same cellular structures in different frames were aligned for parametric measurement and data presentation. This correction was guided by the 405 nm excitation images, which showed no intensity changes during superoxide flashes and were usually omitted from figure presentation for simplicity. Individual superoxide flashes were identified with the aid of a custom computer algorithm, the Flash Sniper [20], and their morphological and kinetic properties, including amplitude (ΔF/F_0_, maximum fluorescence increase over baseline), rise time (RT), full duration at half maximum (FDHM) and full area at half maximum (FAHM) were measured automatically. Two-photon excitation Z-stack images were analyzed using the ZEN 2010 software (Carl Zeiss).

### Histology and Histochemistry

The humeral heads, femoral heads and femoral condyles of the WT and mt-cpYFP transgenic mice were harvested and fixed in 4% formaldehyde. After an overnight wash, the specimens were dehydrated with increasing ethanol concentrations, cleared in xylene and embedded in paraffin, preserving their anatomical orientation. Sections (thickness, 6–7 µm) were cut from paraffin blocks by using a microtome. They were stained with hematoxylin and eosin (H&E) for general histological analysis and with alcian blue for detection of matrix glycosaminoglycan in the articular cartilage according to standard protocols [21]. Histological and histochemically stained sections were observed with a Zeiss Axioplan light microscope system.

### Reagents and Cell Treatments

For labeling mitochondria and nuclei in chondrocytes, articular chondrocytes were incubated with 10 nM TMRM or 5 µg/ml Hoechst 33342 (Molecular Probes) for 30 min at 37°C. Recombinant murine interleukin-1β (IL-1β, PeproTech) and tumor necrosis factor-α (TNF-α, Sigma-Aldrich) were used to induce an inflammatory response. Image acquisition was started immediately after adding IL-1β (10 ng/ml) or TNF-α (10 ng/ml). In some experiments, chondrocytes were pre-incubated with 1 µM cyclosporine A (CsA, Sigma-Aldrich) for 30 min before superoxide flash measurement [18].

### Statistical Analysis

Data have been expressed as mean ± SEM values. The GraphPad Prism version 5.0 statistical software was used to perform two-tailed unpaired Student’s t tests or one-way ANOVAs. Differences were considered statistically significant at *p*<0.05.

## Results

### Mitochondrial Superoxide Flashes in Cultured Articular Chondrocytes

**Figure 1:**
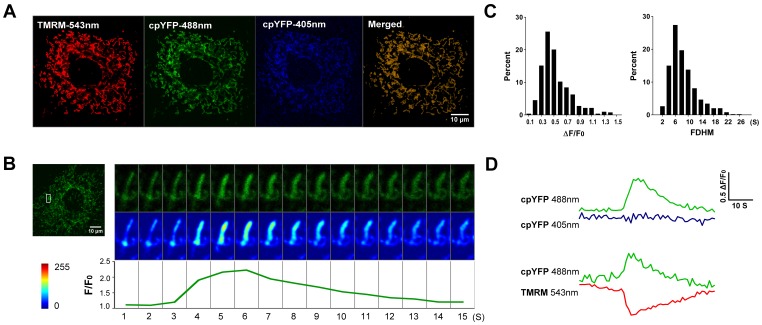
Mitochondrial superoxide flashes in cultured articular chondrocytes. **A**, Colocalization of the superoxide biosensor – mitochondrial circularly permuted yellow fluorescent protein (mt-cpYFP) and tetramethylrhodamine methyl ester (TMRM) in chondrocyte mitochondria revealed by tri-wavelength excitation imaging. Chondrocytes were isolated from mt-cpYFP transgenic mice and cultured as described in the Methods. Only chondrocytes from the first passage were used for experiments. **B**, Mitochondrial superoxide flashes in articular chondrocytes. Enlarged (top panel) and pseudo-color contrast-enhanced (middle panel, scale bar shown to the bottom left) snapshots of a representative mitochondrial superoxide flash in chondrocyte are shown to the right. The lower panel shows the time course of the corresponding mt-cpYFP fluorescence intensity at 488 nm excitation. **C**, Histogram analysis of amplitude (ΔF/F_0_) and full duration at half maximum (FDHM) of chondrocyte superoxide flashes. **D**, Kinetics of superoxide flashes. Note the characteristic lack of fluorescence change at 405 nm excitation. Superoxide flash coincided with transient mitochondrial depolarization (ΔΨ_m_, TMRM at 543 nm excitation).

In order to investigate mitochondrial ROS signaling in articular chondrocytes, we generated a pan-tissue mt-cpYFP transgenic mouse model (see Methods). Histological analysis by hematoxylin/eosin and alcian blue staining did not show any discernible abnormalities in articular cartilage tissue and chondrocyte structures in the transgenic mice (Figure S1). In cultured chondrocytes isolated from the transgenic mice, we stained the mitochondria with TMRM, whose fluorescence signal is spectrally separable from that of mt-cpYFP. Confocal imaging showed that TMRM (excited at 543 nm) and mt-cpYFP (excited at 488 and 405 nm) signals completely overlapped with each other (Figure 1A). The TMRM- and mt-cpYFP-stained mitochondria were relatively uniformly distributed in the cytoplasm, occupying 24.9%±2.7% of the cross-section area of the cell (48 cells). Similarly, TMRM-stained mitochondria in cultured chondrocytes from WT mice occupied 23.9%±4.6% of the cell section (46 cells). Individual mitochondria typically had a filiform shape with lengths ranging from ∼1 to ∼10 µm (Figure S2), but often formed tortuous networks. Time lapse imaging further showed evident mitochondrial motility and dynamic changes in morphological features (Movie S1). These results indicate that mitochondria are abundantly present in articular chondrocytes and may play important functions in these cells.

Figure 1B and Movie S2 show that mitochondria in quiescent cultured chondrocytes undergo spontaneous “superoxide flash” activity [18], which manifest as a sudden quantal burst of superoxide production in single mitochondria. In typical chondrocyte mitochondria, the incidence of superoxide flashes within a 100 s time window of observation was 4.5±0.40 events (n = 115 cells) for a cross-section area of 1000 µm^2^. The average amplitude (ΔF/F_0_) was 0.52±0.01 and the top 10% brightest events showed an amplitude of 1.05±0.02 (n = 51 flashes) which most likely reflects the true amplitude without out-of-focus blurring. A typical chondrocyte flash rose from baseline to peak amplitude in 4.9±0.18 s, showed an FDHM of 8.4±0.19 s, and occupied an area of 0.50±0.02 µm^2^ (FAHM), the size of single mitochondria (n = 511 flashes from 115 cells) (Figure 1C). The distributions of amplitude and kinetic parameters of superoxide flashes in articular chondrocytes (Figure 1C) were also similar to those reported in cardiac myocytes, skeletal muscle cells [19] and cultured hippocampal neurons [18]. Furthermore, discernible mitochondrial swelling occurred during superoxide flashes (Figure 1B). Simultaneous measurement of mt-cpYFP and TMRM showed that every superoxide flash was accompanied with a stable mt-cpYFP signal at 405 nm excitation (an isosbestic point of the indicator) but coincided with a transient decrease in ΔΨm (Figure 1D). Occasionally, we recorded superoxide flashes of complex spatially-extended morphology and displaying conspicuous bead-shaped local swelling (Movie S3). This finding suggests that chondrocyte mitochondria may be organized into convoluted functional networks, as is the case in other cell types [22]–[24]. These results indicate that mitochondria in intact chondrocytes are capable of generating superoxide flashes, which may serve as an elemental mitochondrial ROS signals.

### IL-1β or TNF-α Simulated Superoxide Flash Activity in Cultured Chondrocytes

**Figure 2:**
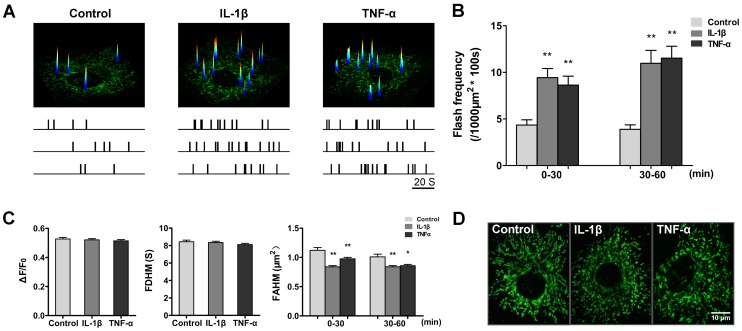
Enhanced superoxide flash activity and mitochondrial fragmentation during IL-1β or TNF-α challenge in cultured chondrocytes. A, Representative examples showing superoxide flash activities in basal conditions and after IL-1β or TNF-α challenges in different chondrocytes. Data are presented as an overlay of the XY view of a chondrocyte (bottom) and surface plot of all superoxide flashes combined from 100 consecutive frames obtained at 1 frame/s. The lower panel shows temporal diaries of superoxide flash incidence in nine representative cells belonging to three groups respectively, with the uppermost diary corresponding to the image shown. B, Superoxide flash frequency without or during IL-1β (10 ng/ml) or TNF-α (10 ng/ml) challenge. Data are shown as the mean ± SEM values. n = 179–576 events from 42–49 cells for each group. **, *p*<0.01 versus the corresponding control group. C, Characteristics of spontaneous (control) and cytokine-stimulated (IL-1β or TNF-α) chondrocytes superoxide flashes. ΔF/F_0_: amplitude. FDHM: full duration at half maximum. FAHM: full area at half maximum. Data represent the mean ± SEM values. n = 42–49 cells for each group. *, *p*<0.05; **, *p*<0.01 versus the corresponding control group. D, Representative chondrocyte mitochondrial morphology before (control) and 60 min after IL-1β or TNF-α treatment.

IL-1β and TNF-α are two main proinflammatory cytokines linked to cartilage destruction [25], [26]. Therefore, we determined the superoxide flash activity in articular chondrocytes during IL-1β or TNF-α challenge to test the effects of proinflammatory cytokines on the production of mitochondrial ROS in chondrocytes. Chondrocytes from the same isolation and passage were treated with IL-1β (10 ng/ml) or TNF-α (10 ng/ml). We found that both IL-1β and TNF-α markedly enhanced superoxide flash activity in cultured chondrocytes (Figure 2A and Movie S4). During the first 30-min treatment, robust 2.2- and 2.0-fold increases in superoxide flash activity were detected for the IL-1β and TNF-α treatment groups, respectively. Elevated superoxide flash activity persisted and even mildly increased during prolonged IL-1β or TNF-α treatment (30–60 min) (Figure 2B). It is noteworthy that ΔF/F_0_ and FDHM did not show statistically significant differences (Figure 2C), indicating that proinflammatory modulation of superoxide flashes occurs mainly in a frequency-modulated manner. Interestingly, the average area of individual flashes decreased from 1.14 µm^2^ (control) to 0.91 µm^2^ (IL-1β) or 0.92 µm^2^ (TNF-α) (Figure 2C) during cytokine stimulation. Consistent with these data, the mitochondria in chondrocytes were progressively fragmented, changing from a mostly filiform to a dominantly punctiform appearance (Figure 2D).

### Imaging Mitochondria in Chondrocytes in situ

**Figure 3:**
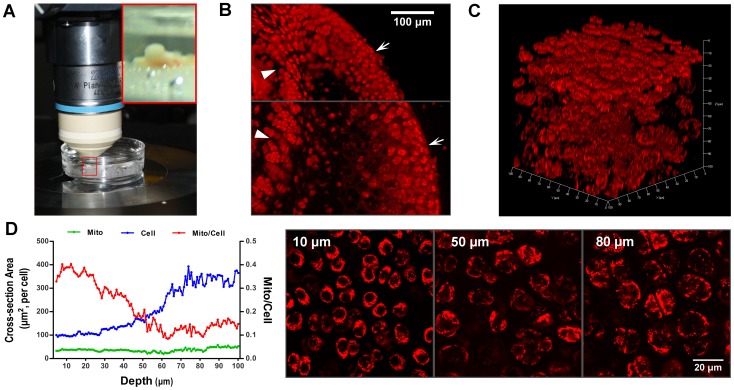
Imaging chondrocytes and mitochondria *in situ*. **A**, Confocal or two-photon-excitation imaging of chondrocyte mitochondria in femoral head cartilage *in situ*. Insert shows an enlarged view of the femoral head. **B**, Mitochondrial distribution in intact cartilage. The femoral head was sliced lengthwise along the midcoronal panel, labeled with TMRM and imaged by a confocal microscope. Arrows mark the cartilage surface and arrowheads mark the growth plate. **C**, Two-photon excitation images of TMRM-stained mitochondria (excitation at 850 nm, red) in chondrocytes at different depths from the cartilage surface. Layer thickness: 0.77 µm, XYZ of 3D: 100*100*100 µm^3^ (Z axis shows depth). **D**, Profiles of averaged mitochondrial (Mito, green) and chondrocyte (Cell, blue) cross-section areas and their ratios (Mito/Cell, red) as a function of distance from the surface. Right panels shows representative images from superficial, middle and deep cartilage zones.

There are great differences between chondrocytes *in situ* and chondrocytes cultured *in vitro* with respect to cell shape and size, oxygen tension and nutrient environment. Using two-photon excitation microscopy, we directly characterized chondrocyte mitochondria in their native environments as in cartilage explants (Figure 3A). In order to study mitochondrial abundance in cartilage at different depths, femoral heads from WT mice were sliced lengthwise along the midcoronal panel and stained with TMRM. The chondrocytes in the articular cartilage (<∼100 µm from the cartilage surface) and epiphyseal cartilage contained abundant mitochondria, while those in calcified cartilage contained only a small amount of mitochondria (Figure 3B). In a different approach, we employed two-photon microscopy and acquired 3D (*xyz*) Z-stack images to resolve mitochondria in living articular chondrocytes in intact layers of femoral head cartilage (Figure 3C and Movie S5). We found that articular chondrocyte density was much lower in middle and deep zones than in superficial zone. Intracellular mitochondrial density (measured using the percentage of cross-section area of the cell) was ∼36% at superficial layer, and showed a precipitous drop in the middle zone (∼28–72 µm), and reached ∼13% in the deep zone of the cartilage (∼72–100 µm). The decrease in mitochondrial density was mainly attributed to the enlargement in cell size from the superficial to the deep zone, because the total cross-section area of mitochondria per chondrocyte was more or less constant across the 100-µm depth (Figure 3D). Thus, mitochondrial distribution in intact cartilage appears to follow a pattern that is apparently related to the microenvironment of chondrocytes in different layers (e.g. oxygen tension and nutrient supply).

### Proinflammatory Cytokines Stimulated Superoxide Flash Activity in situ

**Figure 4:**
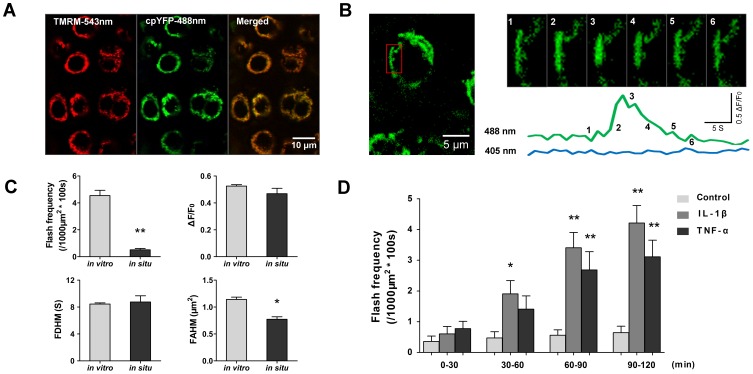
Cytokine-stimulated superoxide flashes in chondrocytes *in situ*. **A** **B**, Time-lapse views of a typical superoxide flash *in situ* in the red boxed region of 2.5 µm×5 µm. Time course plot of the superoxide flash is shown at the bottom. Note the swelling of the mitochondrion during the flash. **C**, Activity and properties of chondrocyte superoxide flashes i*n vitro* (510 events as in Figure. 2) and *in situ* (30 events). Data are reported as the mean ± SEM values. *, *p*<0.05; **, *p*<0.01. **D**, Time course of the superoxide flash response to cytokine stimulation. Data are reported as the mean ± SEM values. n = 21–29 imaging planes. *, *p*<0.05; **, *p*<0.01 versus the respective control.

Confocal imaging showed intense mt-cpYFP expression *in situ* in the humeral head, femoral head and femoral condylar articular cartilage of transgenic mice, colocalizing with the fluorescence of TMRM (Figure 4A). Importantly, we observed robust superoxide flash activity in chondrocytes *in situ,* occurring at a rate of 0.50±0.09 events per 1000 µm^2^ mitochondrial area per 100 s (n = 102 fields of view, 8 mice) (Figure 4B and Movie S6) and showing amplitude and kinetics highly comparable to those of flashes in cultured cells. However, the flash size *in situ* was significantly smaller than *in vitro* (Figure 4C). This finding could be explained by the fact that the chondrocytes *in situ* were of sphere shape with mitochondria randomly oriented relative to the focal plane, but the chondrocytes in culture were flat, with mitochondria extended on the focal plane. That the genesis of superoxide flashes *in situ* was 9-times less active than those in cultured cells may reflect the effect of low-oxygen and low-nutrient on mitochondrial functions (Figure 4C). In addition, it has been shown that culture and monolayer expansion of chondrocytes confers the cells a strong oxidative metabolism and enhances ROS production [27].

The data provided above underscore the necessity to quantify mitochondrial behavior in intact tissue. We therefore re-investigated the effects of proinflammatory cytokines on mitochondrial ROS production in chondrocytes by using the novel *in situ* superoxide flash recording technique. In contrast to the rapid flash response to proinflammatory cytokines, we observed only the trend of enhancement of flash activity in intact cartilage during the early phase of IL-1β or TNF-α challenge (0–30 min), but the increase did not reach statistical significance. We reasoned that dense extracellular matrix could have impeded the diffusion of proinflammatory cytokines. Indeed, we found that the flash activity was elevated by 4.1, 4.8 and 4.9-fold in the continued presence of IL-1β for up to 60, 90 and 120 min, respectively. Similar results were obtained with TNF-α stimulation. As a control, spontaneous flash frequency in the untreated group maintained its low level of activity over the entire period of image acquisition (0–120 min) (Figure 4D). Thus, proinflammatory cytokines can stimulate chondrocytes *in situ*, giving rise to mitochondrial responses in the form of elevated superoxide flash activity.

### Involvement of tMPT in Superoxide Flashes Excited by Proinflammatory Cytokines

**Figure 5:**
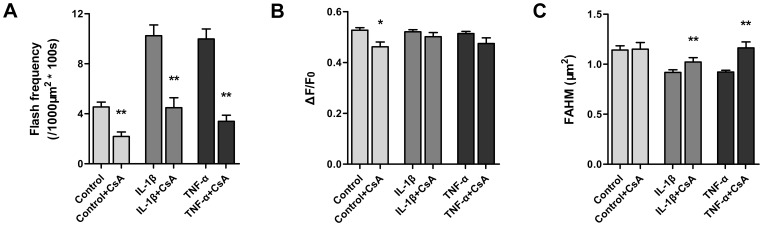
Involvement of tMPT in superoxide flashes excited by proinflammatory cytokines. A, Preincubation of cyclosporine A (CsA, 1 µM for 30 min) suppressed spontaneous superoxide flashes and blunted their response to cytokines. n = 24–115 cells. **B**, Effect of CsA and cytokines on superoxide flash amplitude. **C**, Effect of CsA on cytokine-induced mitochondrial fragmentation, indexed by FAHM. n = 86–969 events. FAHM: full area at half maximum. In **A–C**, data are reported as the mean ± SEM values. *, *p*<0.05; **, *p*<0.01 versus the respective control, IL-1β or TNF-α group.

In articular chondrocytes, conspicuous mitochondrial swelling, masquerading as mitochondrial contraction, often developed with the onset of the superoxide flashes in chondrocytes *in vitro* (Figure 1C) and *in situ* (Figure 4B), supporting the involvement of transient mitochondrial permeability transition (tMPT) in superoxide flash production [18]. Further evidence included concurrent loss of ΔΨ_m,_ monitored by TMRM (Figure 1D). Pharmacological inhibition of mPTP activity by cyclosporine A, which acts on the putative cyclophilin D (CypD) element of the mPTP complex, suppressed mitochondrial superoxide flash activity to 48% of that of the control while also decreasing the amplitude (Figure 5A). Moreover, CsA also attenuated the occurrence of cytokine-induced superoxide flashes, decreased their amplitude (Figure 5B), and protected mitochondria from fragmentation (Figure 5C). Taken together, we conclude that tMPT very likely underlies the trigger of superoxide flashes in quiescent and cytokine-stimulated chondrocytes. In other words, tMPT is a possible previously under-appreciated mediator of the inflammatory response in chondrocytes.

## Discussion

While the structure of the mitochondria in chondrocytes has received some attention from electron microscopic studies [4], [28], the present study used a set of novel *in vitro* and *in situ* optical imaging approaches, and provided rich new information on mitochondrial morphological features, dynamics, distribution, and responsiveness to proinflammatory cytokines in cultured chondrocytes and even in intact cartilage. Mitochondrial abundance as well as chondrocyte density decreased with increase in distance from the surface or marrow cavity, high in the superficial zone but low in the deep zone, high in the growth plate but low in the calcified cartilage. This distribution pattern appears to be in general agreement with decreasing oxygen and nutrient gradients. Moreover, real-time imaging showed that these mitochondria are highly mobile and may be organized into convoluted functional networks, as evidenced by synchronous flashes arising from ignition of complex, presumably interconnected filiform mitochondria.

Increasing evidence indicates that physiological levels of ROS are indispensable in regulating diverse cellular processes [29]. Evidence that oxidants play a supportive role in cartilage ATP synthesis was previously reported by Lee and Urban [3], who demonstrated a negative Pasteur Effect whereby ATP and lactate production was severely impaired under anoxic conditions. It has also been shown that there is a critical need for oxidants that support normal chondrocyte metabolism [10]. With the aid of the novel superoxide biosensor mt-cpYFP and transgenic technology, we found that mitochondria in quiescent chondrocytes exhibit robust superoxide flash activity, with the flash incidence in cultured chondrocytes comparable to that in cultured neuronal and cardiac cells where mitochondria undergo vigorous respiration. However, the flash rate *in situ* was about an order of magnitude lower than that *in vitro*. This might be due to the hypoxic and low-nutrient environments of chondrocytes *in situ* and, perhaps more importantly, the shift of chondrocytes from characteristically glycolytic to oxidative metabolism [27], underscoring the necessity to quantify mitochondrial behavior in intact tissue. Superoxide flashes of articular chondrocytes *in vitro* and *in situ* share many common features, such as a mean amplitude of ∼0.5, time to peak of ∼5 s and half decay time of ∼6 s. In light of the present and previous results, we propose that that mitochondrial superoxide flash production in chondrocytes under physiological conditions represent a house-keeping elemental ROS signaling activity. Mechanistically, concurrent mitochondrial swelling and loss of ΔΨm with superoxide flash production, and sensitivity to CsA all suggest the involvement of the tMPT in the genesis of chondrocyte mitochondrial superoxide flashes, as is the case in many other cell types [18], [24].

Proinflammatory cytokines produced by the inflamed synovial tissue and activated chondrocytes play an important role in the pathophysiology of various cartilage injuries [25], [30]. IL-1β and TNF-α are two of the main cytokines linked to cartilage destruction. They activate the chondrocyte to produce catabolic factors, and are also responsible for inducing chondrocytes apoptosis and other structural changes associated with disease progression [31]. While it has been appreciated that the mitochondrion is one of the most important sites of ROS production in many other cell types [32], the precise contribution of proinflammatory cytokines in mitochondrial alteration in chondrocytes and cartilage degradation remains unknown. To our knowledge, this study is the first to show that proinflammatory cytokines exert a profound effect on the ROS production in chondrocyte mitochondria, in the form of superoxide flashes. We found that after stimulation with IL-1β or TNF-α, mitochondrial superoxide flash production was enhanced by over 2-fold in cultured chondrocytes, and after a 30–60 min delay, nearly 5-fold in chondrocytes *in situ*. The proinflammatory modulation of superoxide flashes occurred mainly in a frequency-coded manner because the amplitude and dynamic parameters remained unchanged.

Superoxide is an unstable “primary” ROS and is readily converted by superoxide dismutase or nonenzymatic chemical reactions into “secondary” ROS [33]. Although it is still unclear whether the production of superoxide in chondrocytes occurs mostly within the mitochondria as many other types of cells [34], the present experiments have demonstrated that mitochondria are active dynamic cellular organelles in living chondrocytes and one of the major sources of superoxide production. Proinflammatory cytokines promote the generation of superoxide by disturbing the normal function of mitochondria and interrupt redox homeostasis at the initial stages. Enhanced oxidative stress can lead to chondrocyte damage and cartilage destruction.

Intracellular ROS production has a close relationship with the dynamic morphological changes of mitochondria [35]. The superoxide flash production was associated with transient mitochondrial swelling (masquerading as mitochondrial “contraction”), and also a progressive persistent mitochondrial fragmentation. The latter has been reported as one of the steps in the process of programmed cell death [36] and as a common and causal event in a variety of degenerative diseases [37]. Our results indicate that the enhancement of superoxide flashes was perhaps the earliest event, followed by mitochondrial fragmentation, in chondrocyte responses to proinflammatory cytokines, well before cytokine-induced activation of the apoptosis pathway which occurs after at least 6 h of stimulation [38]. This finding is in general agreement with recent studies reporting that, in HeLa cells, augmentation of superoxide flash activity constitutes an early signal in oxidative stress-induced apoptosis [24].

In summary, we have shown that mitochondrial superoxide flashes in chondrocytes occur *in vitro* and *in situ* under physiological conditions, suggesting that they are a house-keeping, elemental ROS signaling event. More importantly, proinflammatory cytokine stimulation remarkably increases mitochondrial superoxide flash production and elicits mitochondrial fragmentation. The alternations of chondrocyte mitochondrial morphology and redox balance constitute the earliest chondrocyte responses to inflammatory cytokines and may play an important role in the onset and progression of cartilage inflammatory diseases. In this scenario, targeting these fundamental mitochondrial processes in chondrocytes may hold promise for developing novel and effective therapies for inflammatory cartilage diseases.

## Supporting Information

Figure S1 **Hematoxylin/eosin and alcian staining of femoral condyles, femoral heads and humeral heads from wild type and mt-cpYFP transgenic mice.**
(TIF)

Figure S2 **Mitochondria in cultured articular chondrocytes from a wild type mouse.** Chondrocytes from a wild type mouse were dual-labeled with the mitochondrial dye TMRM (543 nm excitation, red) and the nuclear marker Hoechst 33342 (405 nm excitation, blue) and imaged with a Zeiss LSM 710 microscope.
(TIF)

Movie S1 **Morphological features and motility of mitochondria in living chondrocytes.** This video was produced from 100 time-lapse confocal images of a region of 71×71 µm^2^, collected at a rate of one frame per second, replayed at 60×speeds.
(AVI)

Movie S2 **Superoxide flashes in cultured chondrocytes.** Red arrow denotes a typical superoxide flash. This video was produced from 50 time-lapse confocal images (61×61 µm^2^) collected at a rate of one frame per second, replayed at 20×speed.
(AVI)

Movie S3 **Local swelling of a mitochondrial network undergoing a superoxide flash.** This video was produced from 50 time-lapse confocal images (63×63 µm^2^) collected at a rate of one frame per second, replayed at 20×speed.
(AVI)

Movie S4 **IL-1β-stimulated superoxide flashes in cultured chondrocytes.** This video was produced from 100 time-lapse confocal images (61×61 µm^2^) collected at a rate of one frame per second, replayed at 20×speed.
(AVI)

Movie S5 **Mitochondrial distribution in intact cartilage.** TMRM-stained mitochondria were visualized by two-photon excitation microscopy. The XYZ stack displays a volume of 100*100*100 µm^3^, with Z steps of 0.77 µm and XY pixel size of 0.14 µm.
(AVI)

Movie S6 **IL-1β-stimulated superoxide flashes in chondrocytes** ***in situ*** **.** This video was produced from 100 time-lapse confocal images (25×25 µm^2^) collected at a rate of one frame per second, replayed at 20×speed.
(AVI)
