# Supplementary figures and images for: Proinflammatory Cytokines Stimulate Mitochondrial Superoxide Flashes in Articular Chondrocytes In Vitro and In Situ
Source: PLoS One. 2013 Jun 19;8(6):e66444. doi: 10.1371/journal.pone.0066444 (PMC3686682; doi:10.1371/journal.pone.0066444)

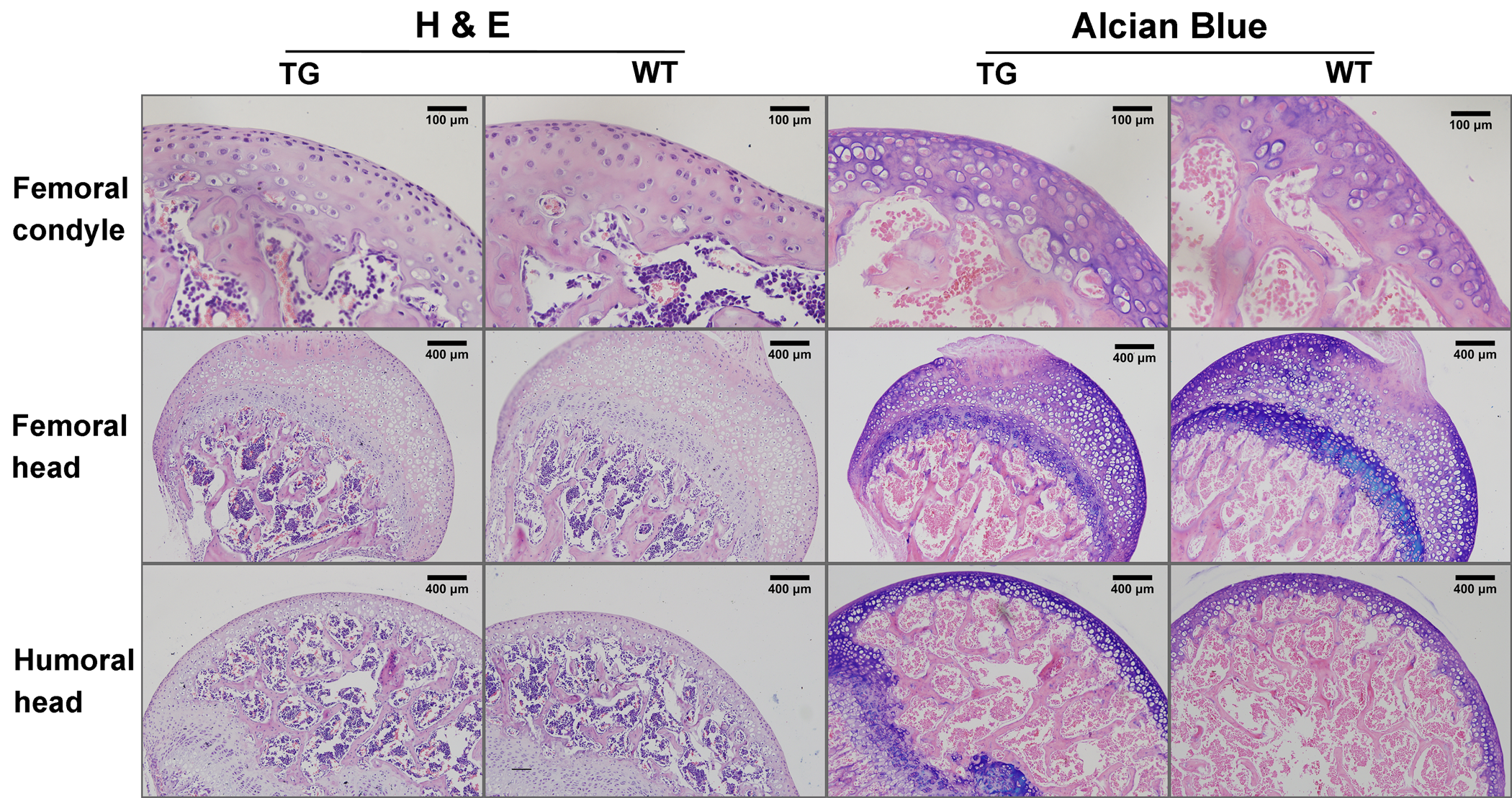

Supplement: Figure S1 — Hematoxylin/eosin and alcian staining of femoral condyles, femoral heads and humeral heads from wild type and mt-cpYFP transgenic mice. (TIF) [file pone.0066444.s001.tif]

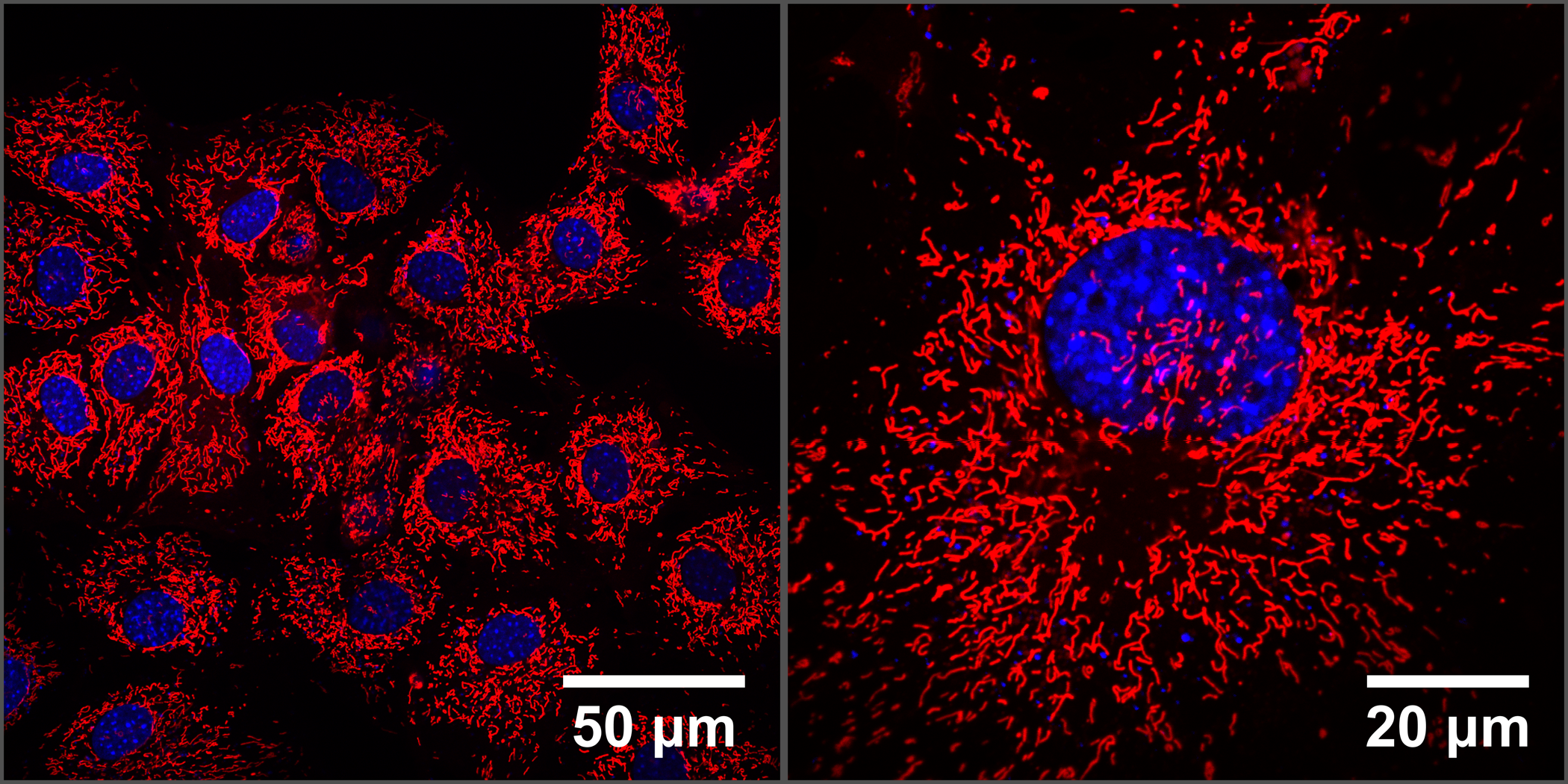

Supplement: Figure S2 — Mitochondria in cultured articular chondrocytes from a wild type mouse. Chondrocytes from a wild type mouse were dual-labeled with the mitochondrial dye TMRM (543 nm excitation, red) and the nuclear marker Hoechst 33342 (405 nm excitation, blue) and imaged with a Zeiss LSM 710 microscope. (TIF) [file pone.0066444.s002.tif]
